# Efficient ammonia production from food by-products by engineered *Escherichia coli*

**DOI:** 10.1186/s13568-020-01083-7

**Published:** 2020-08-18

**Authors:** Yuki Tatemichi, Kouichi Kuroda, Takeharu Nakahara, Mitsuyoshi Ueda

**Affiliations:** 1 Division of Applied Life Sciences, Graduate School of Agriculture, Kyoto University, Sakyo-Ku, Kyoto, 606-8502 Japan; 2 Research and Development Division, Kikkoman Corporation, 338 Noda, Noda-shi, Chiba 278-0037 Japan

**Keywords:** Ammonia, Metabolic profiling, Metabolic engineering, Food by-products, Biorefinery

## Abstract

Ammonia is used as a fertilizer for agriculture, chemical raw material, and carrier for transporting hydrogen, and with economic development, the demand for ammonia has increased. The Haber–Bosch process, which is the main method for producing ammonia, can produce ammonia with high efficiency. However, since it consumes a large amount of fossil energy, it is necessary to develop an alternative method for producing ammonia with less environmental impact. Ammonia production from food by-products is an appealing production process owing to unused resource usage, including waste, and mild reaction conditions. However, when food by-products and biomass are used as feedstocks, impurities often reduce productivity. Using metabolic profiling, glucose was identified as a potential inhibitor of ammonia production from impure food by-products. We constructed the recombinant *Escherichia coli*, in which glucose uptake was reduced by *ptsG* gene disruption and amino acid catabolism was promoted by *glnA* gene disruption. Ammonia production efficiency from okara, a food by-product, was improved in this strain; 35.4 mM ammonia was produced (47% yield). This study might provide a strategy for efficient ammonia production from food by-products.

## Introduction

Ammonia, an important compound in our daily lives, is used as raw material for chemical products such as fertilizers and pharmaceuticals. Ammonia has recently attracted attention as a hydrogen carrier owing to the development of a method for synthesizing hydrogen from ammonia with a fuel cell (Lan et al. 2012; Miura and Tezuka 2014). If the use of ammonia as a liquid carrier of hydrogen gas is promoted in the process of working toward the realization of a hydrogen society, the demand for ammonia will markedly increase. Most of the industrially synthesized ammonia is obtained by the Haber–Bosch process, which yielded approximately 146 million tons in 2018 (James 2019). Haber–Bosch process is very efficient, but the energy and environmental impacts of the process are often a concern.

Food by-products are rich in protein, and some are used in health foods that take advantage of its properties, but most are feed and waste products (Kibler et al. 2018). If a process can be established to efficiently produce ammonia from the amino acids and proteins contained in food by-products, it would not only make better use of unused resources, but could also replace part of the Haber–Bosch process in terms of energy consumption. Food by-products have some advantages, but some issues need to be overcome for industrial use. Several studies have reported the ammonia production from biomass as a by-product of biofuel production using *Escherichia coli* (Huo et al. 2011) or *Bacillus subtilis* (Choi et al. 2014). Therefore, there is still plenty room for improving ammonia production efficiency. In addition, the negative effects of impurities in food by-products on ammonia production should also be considered. Due to the variety of food waste types and the wide range of impurities they contain, it is desirable to rapidly identify productivity-critical impurities, but an efficient process has not been demonstrated.

In recent years, metabolomics has been developed as a tool for understanding life phenomena and applied to the study of microbes (Allen et al. 2003), plants (Fiehn et al. 2000), and mammals (Atherton et al. 2006). Metabolic profiling, developed based on metabolomics technology, can identify critical components with low cost and high throughput (Dunn et al. 2011) and facilitate enhanced comprehension of the relationship between product quality and the chemical components in the product. (Karpe et al. 2015; Shiga et al. 2014). Therefore, metabolic profiling can be used to elucidate the relationship between biomass components and substance production.

In this study, we improved ammonia production from food by-products using metabolic profiling and metabolic engineering to prevent the action of inhibitors. *E. coli* was selected as the host strain for ammonia production due to its potential for efficient ammonia production (Mikami et al. 2017) and ease of genetic modification. Since our experiments of ammonia production from various food by-products suggested the presence of ammonia production inhibitors, we attempted to utilize metabolic profiling to clarify the relationship between the components of food by-products and ammonia production. This approach revealed that glucose in food by-products negatively affected ammonia production. This finding prompted us to engineer *E. coli* to prevent the action of ammonia production inhibitors by disrupting *ptsG* and *glnA*, thereby achieving efficient ammonia production from food by-products.

## Materials and methods

### Strains, plasmids, and media

*E. coli* strains and plasmids used in this study are described in Table 1. Food by-products of soy sauce cake, *mirin* cake, and tomato peel were provided by Kikkoman (Noda, Japan), and okara was purchased from Nippon beans (Isesaki, Japan). The enzymes described below were purchased from Amano-enzyme (Nagoya, Japan). The enzyme mixture was prepared by mixing 20 mg/mL of peptidase ‘ProteAX’, peptidase ‘Peptidase R’, protease ‘Protin SD-AY10′, protease ‘Protease M Amano SD’, protease ‘Protin SD-NY10′, protease ‘Thermoase PC10F’, protease ‘Protease A Amano SD’, hemicellulase ‘Hemicellulase Amano 90’, cellulase ‘Cellulase A Amano 3’, cellulase ‘Cellulase T Amano 4’, mannanase ‘Mannanase BGM Amano 10’ and pectinase ‘Pectinase G Amano’ in 50 mM MES (pH 5.5). To provide an equal amount of the food by-products by dry weight to the pretreatment reaction, a total of 70 g were selected from the following: 3 g soy sauce cake, 3 g *mirin* cake, 9.2 g tomato peel, or 10 g okara (each wet weight) were suspended in MES (pH 5.5) and reacted with 30 mL enzyme mixture at 55 °C for 72 h. The mixture was incubated at 80 °C for 30 min and filtered using ADVANTEC2, AVDANTEC131 (Toyo Roshi Kaisha, Tokyo, Japan), and Millex-HV Syringe Filter Unit, 0.45 μm, PVDF, 33 mm (Merck Millipore, MA, USA). LB broth, YPD broth, M9-yeast extract, M9-tryptone, M9-peptone, and M9-casamino acids (Additional file 1: Table S1) were prepared per previous studies (Matsui et al. 2006; Mikami et al. 2017). Ampicillin (100 μg/mL) (Meiji Seika Pharma, Tokyo, Japan) and kanamycin (25 μg/mL) (Nacalai Tesque, Kyoto, Japan) were added as appropriate.

**Table 1:** *E. coli* strains and plasmids used in the study

Strains/plasmids	Description	Source
*E. coli* strains		
DH10B	F^-^ *mcrA*Δ(*mrr*-*hsdRMS*-*mcrBC*) φ80d *lacZ*ΔM15 Δ*lacX74 recA1 endA1 araD139* Δ(*araleu*)7697 *galU galK*^-^ *rpsL nupG*	Thermo fisher
DH10B (pKD46)	DH10B harboring pKD46	Mikami et al. (2017)
∆*gln*A	DH10B, Δ*glnA*::FRT-kan^R^-FRT	Mikami et al. (2017)
∆*pts*G	DH10B, Δ*ptsG*::FRT-kan^R^-FRT	This study
∆*pts*G-*kan*	DH10B, Δ*ptsG*::FRT	This study
∆*pts*G∆*gln*A	DH10B, Δ*ptsG*::FRT Δ*glnA*::FRT-kan^R^-FRT	This study
Plasmids		
pKD46	Red recombinase expression plasmid	Datsenko et al. (2000)
pKD13	Kan^R^ template plasmid	Datsenko et al. (2000)
pCP20	Temperature-sensitive replication and thermal induction of FLP synthesis	Cherepanov and Wackernagel (1995)

### Construction of *E. coli* strains

Gene deletion mutants were constructed using the previously reported method (Datsenko and Wanner 2000). A *ptsG*-Km-resistant fragment was amplified from pKD13 (Datsenko and Wanner 2000) through polymerase chain reaction (PCR) with primers ptsGF and ptsGR containing homologous sequences upstream and downstream of the *ptsG* coding region and flippase recognition target (FRT) sequence (Additional file 1: Table S2). *E. coli* DH10B (pKD46) was electroporated with the *ptsG*-Km resistant fragment and plated on LB agar containing ampicillin and kanamycin at 30 °C. After incubation at 37 °C to eliminate the temperature-sensitive plasmid pKD46, the resulting ampicillin-sensitive strain (*ptsG*::FRT-*kan*^R^-FRT) was designated ∆*ptsG*. ∆*ptsG*-kan strain was constructed from ∆*ptsG* using flippase (FLP) helper plasmid pCP20 (Datsenko and Wanner 2000). A *glnA*-Km resistant fragment was amplified from pKD13 using PCR with primers glnAF and glnAR containing homologous sequences upstream and downstream of the *glnA* coding region and FRT sequence (Additional file 1: Table S2). Strains, ∆*glnA* and ∆*ptsG*∆*glnA*, were constructed from DH10B and ∆*ptsG* respectively, using the *glnA*-Km resistant fragment. To confirm the desired genome insertion of the resistance fragments, the insertion region was amplified by PCR and sequenced using insertion-checking primers (Additional file 1: Table S2).

### Evaluation of ammonia production and glucose uptake

Ammonia production experiments were conducted as previously described (Mikami et al. 2017). *E. coli* strains were initially grown overnight in M9-yeast extract, then the cells were washed with sterile water and inoculated into 2 mL of pretreated food by-products or semisynthesized medium to a final OD_600_ of 0.5. After incubation at 37 °C for 26.5 h with shaking, the concentrations of ammonia in the supernatants were measured by F-kit ammonia (J.K. International, Tokyo, Japan). To evaluate the glucose uptake, each of the strains cultured overnight in M9-yeast extract was added to 50 mM glucose with OD_600_ of 0.5 and incubated for 2 h. After centrifugation at 5000 *g*, 4 °C for 5 min, the supernatant was collected and diluted with water five times. The amount of residual glucose was measured using the glucose CII-test-Wako (Wako, Osaka, Japan) to evaluate glucose uptake.

### Quantification of ammonia, amino acids, organic acids, and sugars in medium and culture supernatant

The medium and culture supernatants were filtered using a 0.45 μm DISMIC filter (Advantec Toyo, Tokyo, Japan). The amino acid analysis was conducted on an L-8900 amino acid analyzer (Hitachi, Tokyo, Japan). Organic acids were quantified with an ST-3 (Showa Denko, Tokyo, Japan) post-column reaction system (Sano et al. 2007; Wada et al. 1984) using prominence high-performance liquid chromatography (HPLC) (Shimadzu, Kyoto, Japan) equipped with an RSpak KC-811 column (300 mm × 8.0 mm, 6 μm; Showa Denko, Tokyo, Japan). Analysis of sugars was conducted using the phenylhydrazine post-column method (Suzuki et al. 2009) by employing a chromstar 5510 (Hitachi, Tokyo, Japan) equipped with an Asahipak NH2P-50 4E column (4.6 mm × 250 mm) (Showa Denko, Tokyo, Japan).

### Statistics

The statistical screening was conducted by metabolic profiling (Shiga et al. 2014) with modifications as described briefly below. Partial least squares (PLS) regression analysis was carried out with SIMCA-P software (version 12.0, Umetrics, Umeå, Sweden). The data of ammonia production and its medium components were analyzed using Pareto scaling and PLS regression analysis model. The variable importance in the projection (VIP) (Eriksson et al. 2006) and regression coefficient were calculated to select candidate inhibitors of ammonia production. The VIP_Ak_ value of the k_th_ explanatory variable in K variables of PLS with the A_th_ component was calculated using the following equation:

$${\text{VIP = }}\sqrt {\mathop \sum \limits_{\text{a = 1}}^{\text{A}} \left( {{\text{w}}_{\text{ak}}^{ 2} \times \left( {{\text{SSY}}_{\text{a - 1}} {\text{ - SSY}}_{\text{a}} } \right)} \right) \times \frac{\text{K}}{{ ( {\text{SSY}}_{ 0} {\text{ - SSY}}_{\text{A}} )}}}$$

A is the total of the latent variable, w_ak_ is the PLS weight of the k_th_ variable in the a_th_ latent variable, and SSY indicates the variance of the predicted residuals by each PLS component. The VIP value indicates the contribution of each explanatory variable to the model (Eriksson et al. 2006). The “VIP scores > 1” rule is generally used as the criterion for important variable selection (Afanador et al. 2013). The variable, *Y*, the variable, *X*, the coefficients, *B*, and error matrix, F*, are represented by the internal relationship *Y* = *XB* + F* (Palermo et al. 2009). For interpreting the influence of the variables *X* on *Y,* the coefficients are calculated as coeffCS in SIMCA-P (Eriksson et al. 2006). Dunnett’s and Tukey’s tests were performed using the JMP software version 14 (SAS, North Carolina, U.S.) for multiple comparisons.

## Results

### Screening for inhibitors of ammonia production from pretreated food by-products

**Fig. 1:**
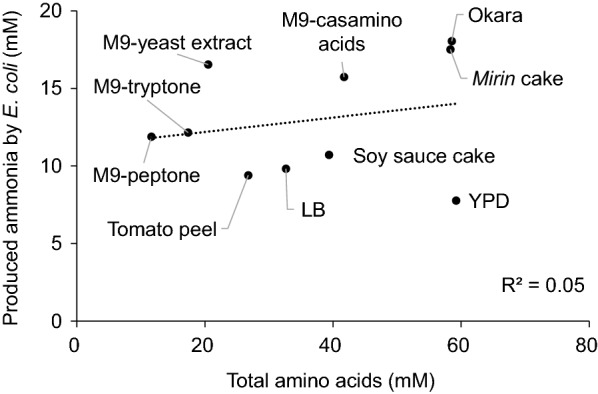
Relationship between ammonia production and total amino acids. Ammonia was produced by *E. coli* DH10B from four food by-products and six media containing amino acids. *E. coli* strains were initially grown overnight in M9-yeast extract, then the cells were washed with sterile water and inoculated into 2 mL of pretreated food by-products or semisynthesized medium to a final OD_600_ of 0.5. After incubation at 37 °C for 26.5 h with shaking, the concentrations of ammonia in the supernatants were measured. The data of ammonia concentration are represented as the mean values (n = 3), and total amino acid concentration in the medium was determined at n = 1

The amount of ammonia produced from food by-products should correlate with the available amino acids, a major nitrogen source in food by-products, because the amino acid concentration in the medium affects *E. coli* ammonia production. (Jeremy et al. 2002; Mikami et al. 2017). However, ammonia production could be inhibited by other components present in food by-products. To examine whether inhibitory components of ammonia production are present in food by-products, ammonia was produced by an *E. coli* wild-type strain (DH10B) from four pretreated food by-products and six media containing rich amino acids. Ammonia production was not correlated with the concentration of total amino acids in the media (R^2^ = 0.05, Fig. 1), suggesting that the pretreated food by-products contain potent ammonia production inhibitors.

**Fig. 2:**
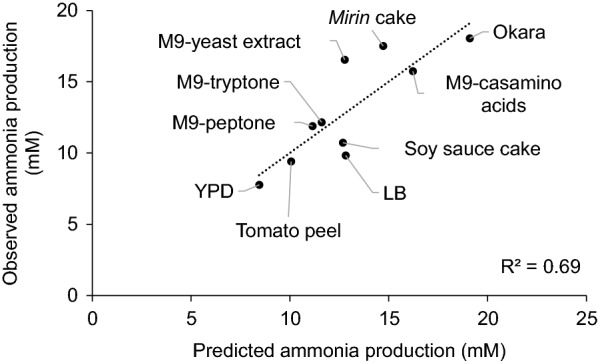
Relationship between actual and predicted ammonia production using the PLS model. The concentration of ammonia produced by *E. coli* DH10B from four food by-products and six media containig amino acids was plotted against the concentration of ammonia predicted by PLS regression. The data of ammonia concentration are expressed as the mean values (n = 3), and the predicted ammonia production was calculated by PLS regression analysis using the data (n = 1) of the concentration of amino acids, sugars, and organic acids in the medium

Metabolic profiling by PLS regression analysis was then performed to identify the inhibitors. VIP values obtained by PLS regression analysis can be used to predict important factors in a wide range of fields, including epidemiological studies (Jonasson et al. 2007; Yan et al. 2019), food science (Harsha et al. 2018; Shiga et al. 2014), and biotechnology (Chen et al. 2018; Zalai et al. 2015). To clarify the relationship between metabolite data and ammonia production, PLS regression and VIP analysis were performed using analytical data for amino acids, sugars, and organic acids as explanatory variables and ammonia production as response variables. Figure 2 shows the predicted value and the measured value of ammonia production. The correlation of the measured amount of ammonia production with the amount predicted by PLS regression (R^2^ = 0.69) was better than that with the amount of total amino acids (R^2^ = 0.05).

The VIP and regression coefficient were used to detect the inhibitor. VIP is an indicator of the effect of explanatory variables on model response variables and is used to select important metabolites in metabolomics (Fonville et al. 2010). The regression coefficient determines whether an explanatory variable has a positive or negative effect on a response variable. Glucose showed the highest VIP with a negative regression coefficient, indicating that glucose is the most probable ammonia production inhibitor (Table 2). In bioethanol production, glucose concentrations up to 2.78 M are produced from food by-product digests (Mohd Azhar et al. 2017). In contrast, pretreated food by-products, which are mainly composed of amino acids, have a lower glucose concentration than biomass digests (74 mM to 123 mM) (Additional file 1: Table S1 and S3).

**Table 2:** Compound selected by variables in the projection (VIP) analysis

Compound	VIP	Coefficient
Glucose	2.11	− 1.52
Isomaltose	2.09	2.89
Galactose	2.07	2.76
Arabinose	1.48	1.97
Proline	1.36	1.79
Glutamine	1.16	1.62
Fructose	1.06	− 1.41
Glutamic acid	1.06	1.39
Serine	1.04	1.43

**Fig. 3:**
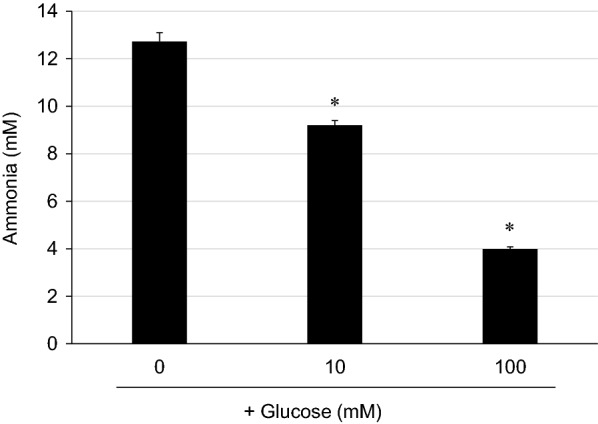
Inhibitory effect of glucose on ammonia production by *E. coli* DH10B. *E. coli* DH10B was incubated in M9-yeast extract with or without glucose (10 or 100 mM) for 26.5 h to produce ammonia. Values are expressed as the mean ± SD (n = 3). * Statistical significance was determined by Dunnett’s test (P < 0.001)

To confirm whether glucose is a true inhibitor of ammonia production from food by-products, the effect of glucose addition on ammonia production was examined in an M9-yeast extract medium. Ammonia production was inhibited in a glucose concentration-dependent manner and decreased to 31% of that before glucose was added (Fig. 3). Furthermore, the quantification of the remaining amino acids in the supernatant indicated that the consumption of eight amino acids decreased in a glucose concentration-dependent manner (Additional file 1: Figure S1). These results suggest that glucose inhibits ammonia production from pretreated food by-products by inhibiting amino acid cellular metabolism.

### Gene deletion of *E. coli* to improve ammonia production

Based on the above experimental results, we attempted to develop *E. coli* for more efficient ammonia production from pretreated food by-products. To avoid the negative effects of glucose, we suppressed the glucose uptake function in *E. coli* by impairing the phosphoenolpyruvate sugar phosphotransferase system (PTS) that transports major sugars, such as glucose. PTS plays an important role in favoring glucose uptake over other carbon sources (Postma et al. 1993). PTS consists of a phosphohistidine carrier protein, an enzyme I component, and enzymes EIIA, EIIB, and EIIC, which are single polypeptide chains encoded by *ptsG* (Escalante et al. 2012). It was reported that the inactivation of *ptsG* reduces glucose uptake efficiency (Steinsiek and Bettenbrock 2012). Therefore, we disrupted *ptsG* to impair the PTS in *E. coli.* An additional gene, *glnA* encoding glutamine synthetase, was also disrupted to promote the conversion of amino acids to ammonia because a previous study (Mikami et al. 2017) showed that ammonia production during incubation in M9-yeast extract is greatly increased by disrupting *glnA* in *E. coli*.

**Fig. 4:**
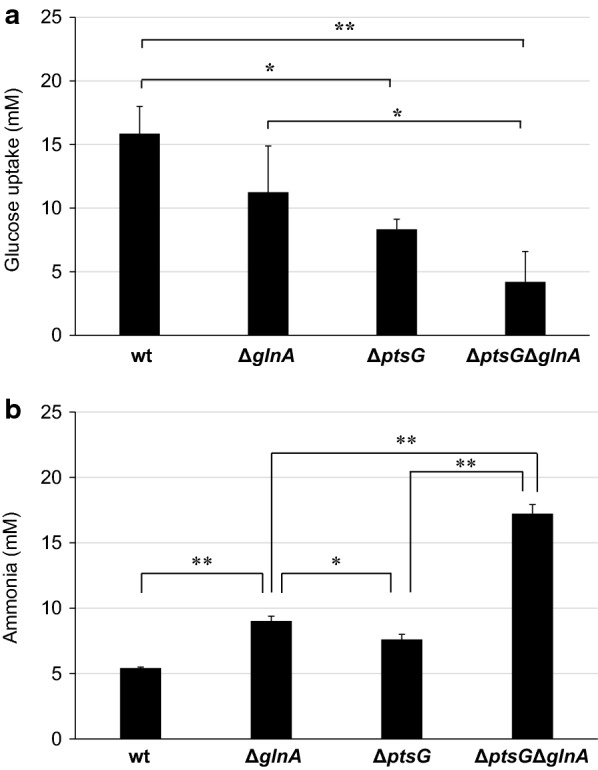
Effects of *ptsG* and *glnA* disruption on glucose uptake (**a**) and ammonia production (**b**) in the M9-yeast extract medium with 50 mM glucose. Glucose uptake and ammonia production was measured 2 h and 26.5 h after the start of incubation at 37 °C in the M9-yeast extract medium, respectively. Values are expressed as the mean ± SD (n = 3). Statistical significance was determined by Tukey’s test (*P < 0.05, **P < 0.01)

To investigate whether suppression of glucose uptake and synthesis of glutamine by glutamine synthetase improves ammonia production from glucose-containing media, we performed ammonia production by Δ*ptsG*, Δ*glnA*, and Δ*ptsG*Δ*glnA* strains. In an M9-yeast extract medium containing 50 mM glucose, Δ*ptsG* and Δ*ptsG*Δ*glnA* strains showed lower glucose uptake rates than the wild-type and Δ*glnA* strains (Fig. 4a). Furthermore, these strains showed higher ammonia productivity than the wild-type strain (Fig. 4b). In particular, Δ*ptsG*Δ*glnA* strain produced about 3.2 fold more ammonia than the wild-type strain. These results suggest that the disruptions of *ptsG* and *glnA* can improve ammonia production even in the medium containing high concentrations of glucose, such as food by-products.

### Ammonia production from pretreated food by-products by engineered *E. coli*

To evaluate the ammonia productivity of the engineered *E. coli* strains (Δ*ptsG*, Δ*glnA*, and Δ*ptsG*Δ*glnA*), these strains were utilized to produce ammonia from okara, a model of food by-products. Okara is the residue produced in soy milk and tofu production; 1.1 kg of wet okara is produced from 1 kg of soybeans and water (Khare et al. 1995). Due to high protein concentrations (27% to 38%), most of the produced okara is used as animal feed or discarded (Surel and Couplet 2005). The proteins included in okara are water-insoluble and cumbersome but can be efficiently extracted by hydrolyzing the cell wall with cellulase (de Figueiredo et al. 2018). Accordingly, the extracted proteins were treated with protease to produce amino acids, which can be a nitrogen source for ammonia production. We obtained 58.5 mM free amino acids by solubilizing okara with cellulase and protease (Additional file 1: Table S1).

**Fig. 5:**
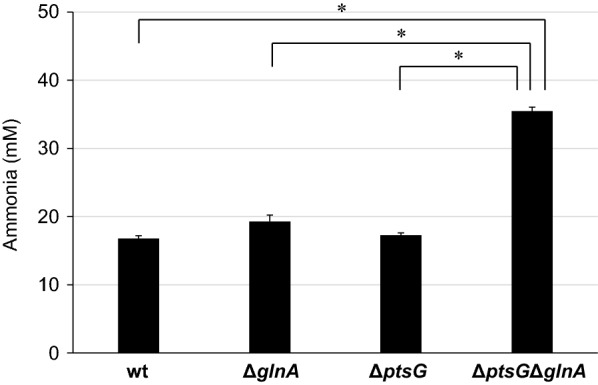
Ammonia production from pretreated okara by engineered *E. coli* strains. Ammonia was produced by incubating cells in the pretreated okara solution at 37 °C for 26.5 h. Values are expressed as the mean ± SD (n = 3). Statistical significance was determined by Tukey’s test (*P < 0.0001)

The pretreated okara was then incubated with the engineered *E. coli* for ammonia production. The Δ*ptsG* and Δ*glnA* strains slightly increased ammonia production compared with the wild-type strain, suggesting that the strategies for disruption of glucose transporters and the promotion of amino acid catabolism are also effective for ammonia production from the pretreated okara (Fig. 5). Furthermore, the Δ*ptsG*Δ*glnA* strain, which had the highest ammonia production efficiency in the M9-yeast extract containing 50 mM glucose, also exhibited the highest ammonia production efficiency in the pretreated okara. This represents a 2.1-fold improvement over the wild type, with a conversion efficiency from amino acids of about 47%. These results indicate that the combination of reducing glucose uptake and promoting amino acid catabolism is an effective strategy of efficient ammonia production from pretreated food by-products.

## Discussion

The effective use of food by-products is expected to become increasingly important, considering the extensive efforts required for the realization of a sustainable society (Avtar et al. 2019). In this study, we attempted to screen ammonia production inhibitors included in pretreated food by-products using metabolic profiling. The biomarker screen using VIP and coefficients calculated from PLS regression analysis has been widely used (Jonasson et al. 2007; Yan et al. 2019; Harsha et al. 2018; Shiga et al. 2014; Chen et al. 2018; Zalai et al. 2015), and it was also effective in our screening method for inhibitors from food by-products. Glucose has been known to exert important effects on amino acid metabolism, but this is the first study to highlight the negative effects of glucose on ammonia production from food by-products. Based on this finding, the recombinant *E. coli* with high ammonia productivity was successfully constructed.

Our experiments showed that glucose in food by-products suppressed ammonia production by inhibiting the metabolism of several amino acids (Fig. 2). Ammonia metabolism is tightly regulated in *E. coli*, with the major operon *glnALG* encoding the glutamine synthetases, nitrogen regulatory protein B (NtrB), and nitrogen regulatory protein C (NtrC) (Reitzer and Magasanik 1986). Transcription of the *glnALG* operon is regulated by tandem promoters *glnA*p1 and *glnA*p2. *glnA*p1 is a σ70 dependent promoter that is activated by the cyclic AMP receptor protein (CRP) under high glucose conditions and repressed by NtrC-phosphate under nitrogen-deficient conditions. *glnA*p2 is a σ54 dependent promoter that is strongly activated by NtrC-phosphate and repressed by CRP (Tian et al. 2001). Glutamine synthetase is regulated by the *glnA*p1 promoter and activated through CRP when glucose is added to M9-yeast extract. This may promote ammonia assimilation and lead to a decrease in free ammonia.

Additionally, the metabolism of amino acids, especially Gly, was inhibited by the M9-yeast extract containing 50 mM glucose (Additional file 1: Figure S1). This was possibly due to the inhibition of purine nucleotide synthesis repressor gene (*purR*) translation by small regulatory RNA (SgrS) in response to glucose stress (Bobrovskyy and Vanderpool 2016; Vanderpool and Gottesman 2004). Since *purR* inhibits the expression of glycine cleavage system proteins encoded by *gcvTHP* that induces Gly degradation (Cho et al. 2011), there is a possibility that ammonia production by Gly degradation was inhibited under glucose-added conditions.

To suppress ammonia assimilation and promote amino acid metabolism, two strategies were developed to reduce glucose uptake and inhibit glutamine synthetase. To reduce glucose uptake, we disrupted *ptsG*, which encodes a major component of the glucose transporter. However, glucose is also taken up into *E. coli* cells though other transporters such as the galactose transporter (encoded by *mglBAC*), galactose permease (encoded by *galP*), and maltose transporter (encoded by *malEFG*) (Death and Ferenci 1993; Ferenci 1996). In fact, the ammonia production efficiency from *mirin* cake containing excess glucose (123.3 mM) by the *ΔptsGΔglnA* strain was 23%, which was lower than that from okara (47%). By disrupting these transporters simultaneously, glucose uptake efficiency could be further reduced, and ammonia production efficiency could be further improved.

A single disruption of *glnA* improved ammonia production from glucose-added M9-yeast extract, while dual disruption of *ptsG* and *glnA* markedly improved ammonia production efficiency (Fig. 4b). In Δ*ptsG*, inhibition of glucose uptake might lead to a decrease in CRP and activation of *glnA*p2. This activates NtrC and nitrogen assimilation control (Nac) proteins, which control approximately 2% of the genes involved in nitrogen catabolism (Zimmer et al. 2000), resulting in the increased production of free ammonia. However, the produced ammonia can be assimilated by glutamine synthetase into amino acids in the cells. Additional disruption of *glnA,* which encodes glutamine synthetase, would increase the free ammonia concentration by inhibiting the assimilation of ammonia to glutamine in the Δ*ptsG* strain, resulting in a synergistic improvement in ammonia productivity. Disrupting genes involved in ammonia assimilation, such as *gdhA* and *gltBD*, is expected to further improve ammonia productivity, but there are concerns about growth retardation and lethality issues. In fact, growth delays have been observed in *ΔglnA* and *ΔglnAΔptsG*, but knockdown of the ammonia assimilation genes by CRISPRi (Hawkins et al. 2015; Qi et al. 2013) or other methods could solve these problems.

The Δ*ptsG*Δ*glnA* strain produced 35.4 mM ammonia from 58.5 mM amino acids in the pretreated okara (Fig. 5). At the end of incubation, the ammonia production rate was 1.34 mM/h and the conversion efficiency from amino acids to ammonia was approximately 47%, and further improvement of the efficiency is required for industrial use. This result was lower than the conversion efficiency of approximately 73% in the M9-yeast extract containing 50 mM glucose (Fig. 4b), suggesting that other ammonia production inhibitors were present in the pretreated okara to suppress amino acid metabolism or promote ammonia assimilation. To improve ammonia productivity, monitoring of amino acid metabolism by amino acid flux analysis, large-scale analysis by LC–MS and GC–MS, identification of novel inhibitors, and subsequent genetic modification are needed.

In this study, we identified glucose as the cause of inhibitory effects on ammonia production from food by-products using an efficient screening strategy. We improved ammonia production by *E. coli* through the reduction of glucose uptake and the promotion of amino acid catabolism by gene disruptions. These findings provide important insights into ammonia production in *E. coli* as well as an industrially available strain (e.g., *B. subtilis and S. cerevisiae*) and would contribute to the study of ammonia production from various biomasses or other unused resources, potentially expanding the application of ammonia bioproduction.

## Supplementary information

**Additional file 1: Table S1.** Concentration of glucose in various medium. **Table S2**. Primers used in this study. **Table S3.** Concentration of amino acids in various medium (expressed as mM). **Figure S1.** M9-yeast extract medium supplemented with (A) blank, (B) 10 mM glucose, or (C) 50 mM glucose was incubated by *E. coli *DH10B for 1–26.5 hours, and the amino acid concentrations were compared. Values are given as mean ± SD (n = 3).

## Data Availability

All relevant data are within the manuscript and its Additional file 1.

## Abbreviations

*E. coli*: *Escherichia coli*
PCR: Polymerase chain reaction
HPLC: High-performance liquid chromatography
PLS: Partial least squares
VIP: Variable importance in the projection
PTS: Phosphotransferase system
CRP: Cyclic AMP receptor protein
Nac: Nitrogen assimilation control
