# Supplementary material for: Efficient ammonia production from food by-products by engineered Escherichia coli
Source: AMB Express. 2020 Aug 18;10:150. doi: 10.1186/s13568-020-01083-7 (PMC7434829; doi:10.1186/s13568-020-01083-7)
Supplement: Supplementary file 1 — Additional file 1: Table S1. Concentration of glucose in various medium. Table S2. Primers used in this study. Table S3. Concentration of amino acids in various medium (expressed as mM). Figure S1. M9-yeast extract medium supplemented with (A) blank, (B) 10 mM glucose, or (C) 50 mM glucose was incubated by E. coli DH10B for 1–26.5 hours, and the amino acid concentrations were compared. Values are given as mean ± SD (n = 3). [file 13568_2020_1083_MOESM1_ESM.pdf]

**Table S1 Concentration of glucose in various medium**

| Medium | Type | Glucose (mM) | Fructose (mM) | Total amino acids (mM) |
|---|---|---|---|---|
| Soy sauce cake | Food by-product | 88.6 | 3.1 | 39.4 |
| *Mirin* cake | Food by-product | 123.3 | 2.3 | 58.3 |
| Tomato peel | Food by-product | 95.7 | 4.0 | 26.8 |
| Okara | Food by-product | 74.4 | 12.9 | 58.5 |
| M9-yeast extract | Semisynthesized medium | n.d. | n.d. | 20.5 |
| M9-tryptone | Semisynthesized medium | n.d. | n.d. | 17.4 |
| M9-peptone | Semisynthesized medium | n.d. | n.d. | 11.6 |
| M9-casamino acids | Semisynthesized medium | n.d. | n.d. | 41.7 |
| LB | Semisynthesized medium | n.d. | n.d. | 32.6 |
| YPD | Semisynthesized medium | 108.6 | n.d. | 59.2 |

n.d.: not detected

**Table S2 Primers used in this study**

| Primer | Sequence (5′-3′) | Note |
|---|---|---|
| glnAF | GCCAGAGACAGGCGAAAAGTTTCCACGGCA-ACTAAAACACGTGTAGGCTGGAGCTGCTTC | Construction for *glnA*-Km cassette |
| glnAR | GTTACCACGACGACCATGACCAATCCAGGAG-AGTTAAAGTCTGTCAAACATGAGAATTAA | Construction for *glnA*-Km cassette |
| ptsGF | AACGTAAAAAAAGCACCCATACTCAGGAGC-ACTCTCAATTCTGTCAAACATGAGAATTAA | Construction for *ptsG*-Km cassette |
| ptsGR | CAGCCATCTGGCTGCCTTAGTCTCCCCAACG-TCTTACGGAGTGTAGGCTGGAGCTGCTTC | Construction for *ptsG*-Km cassette |
| glnAseqF | AGCTGACAAACTTCACGTTG | Check the genome insertion of *glnA*-Km cassette |
| glnAseqR | GCAACATTCACATCGTGGTG | Check the genome insertion of *glnA*-Km cassette |
| ptsGseqF | GTCAAACAAATTGGCACTG | Check the genome insertion of *ptsG*-Km cassette |
| ptsGseqR | CAATAGCAGCCAGTCCCTTC | Check the genome insertion of *ptsG*-Km cassette |

**Table S3 Concentration of amino acids in various medium (expressed as mM)**

| | Soy sauce cake | *Mirin* cake | Tomato peel | Okara | M9-yeast extract | M9-tryptone | M9-peptone | M9-casamino acids | LB | YPD |
|---|---|---|---|---|---|---|---|---|---|---|
| Aspartic acid | 1.91 | 2.71 | 1.85 | 2.85 | 1.17 | 0.42 | 0.29 | 3.74 | 1.19 | 2.56 |
| Threonine | 2.50 | 3.46 | 1.80 | 3.61 | 1.05 | 1.02 | 0.39 | 2.45 | 1.62 | 2.86 |
| Serine | 2.86 | 4.40 | 1.98 | 4.62 | 1.40 | 0.87 | 0.46 | 3.37 | 1.80 | 3.16 |
| Glutamic acid | 2.94 | 4.40 | 2.70 | 6.08 | 3.64 | 0.95 | 0.75 | 10.38 | 3.46 | 7.31 |
| Glycine | 2.10 | 3.54 | 1.40 | 3.34 | 1.23 | 0.36 | 1.28 | 1.76 | 1.18 | 5.09 |
| Alanine | 3.73 | 5.55 | 2.43 | 4.95 | 3.43 | 1.01 | 1.30 | 2.67 | 3.36 | 8.84 |
| Cysteine | 0.41 | 0.98 | 0.39 | 0.80 | n.d. | n.d. | n.d. | n.d. | n.d. | n.d. |
| Valine | 3.11 | 5.18 | 2.08 | 4.64 | 1.69 | 1.59 | 0.83 | 3.65 | 2.85 | 4.73 |
| Methionine | 0.40 | 1.20 | 0.35 | 0.71 | 0.40 | 0.33 | 0.13 | 0.76 | 0.89 | 0.17 |
| Isoleucine | 2.18 | 3.26 | 1.34 | 3.21 | 1.20 | 0.87 | 0.43 | 1.88 | 1.73 | 2.89 |
| Leucine | 3.16 | 5.31 | 2.17 | 4.97 | 2.01 | 3.29 | 1.11 | 3.15 | 4.96 | 5.97 |
| Tyrosine | 5.48 | 2.92 | 1.33 | 2.30 | 0.39 | 0.25 | 0.26 | 0.22 | 0.51 | 1.17 |
| Phenylalanine | 2.04 | 2.80 | 1.11 | 2.59 | 0.96 | 1.49 | 0.54 | 1.34 | 2.24 | 2.86 |
| Lysine | 1.68 | 1.84 | 1.61 | 3.14 | 0.06 | 0.03 | n.d. | 0.02 | 0.06 | 0.22 |
| Histidine | 0.48 | 0.72 | 0.39 | 0.96 | 0.09 | n.d. | 0.03 | n.d. | 0.13 | 0.16 |
| Arginine | 1.15 | 2.44 | 1.08 | 2.69 | 0.67 | 2.54 | 0.95 | 3.21 | 3.34 | 3.32 |
| Proline | 1.46 | 2.67 | 0.93 | 2.85 | 0.19 | 0.31 | 0.11 | 0.47 | 0.39 | 0.56 |
| Asparagine | 1.30 | 2.52 | 1.21 | 2.83 | 0.20 | 0.24 | 0.13 | 0.97 | 0.49 | 0.34 |
| Glutamine | 0.48 | 2.42 | 0.61 | 1.41 | 0.70 | 1.78 | 2.64 | 1.68 | 2.45 | 7.01 |
| Total | 39.4 | 58.3 | 26.8 | 58.5 | 20.5 | 17.4 | 11.6 | 41.7 | 32.6 | 59.2 |

n.d.: not detected

**Fig.S1A**

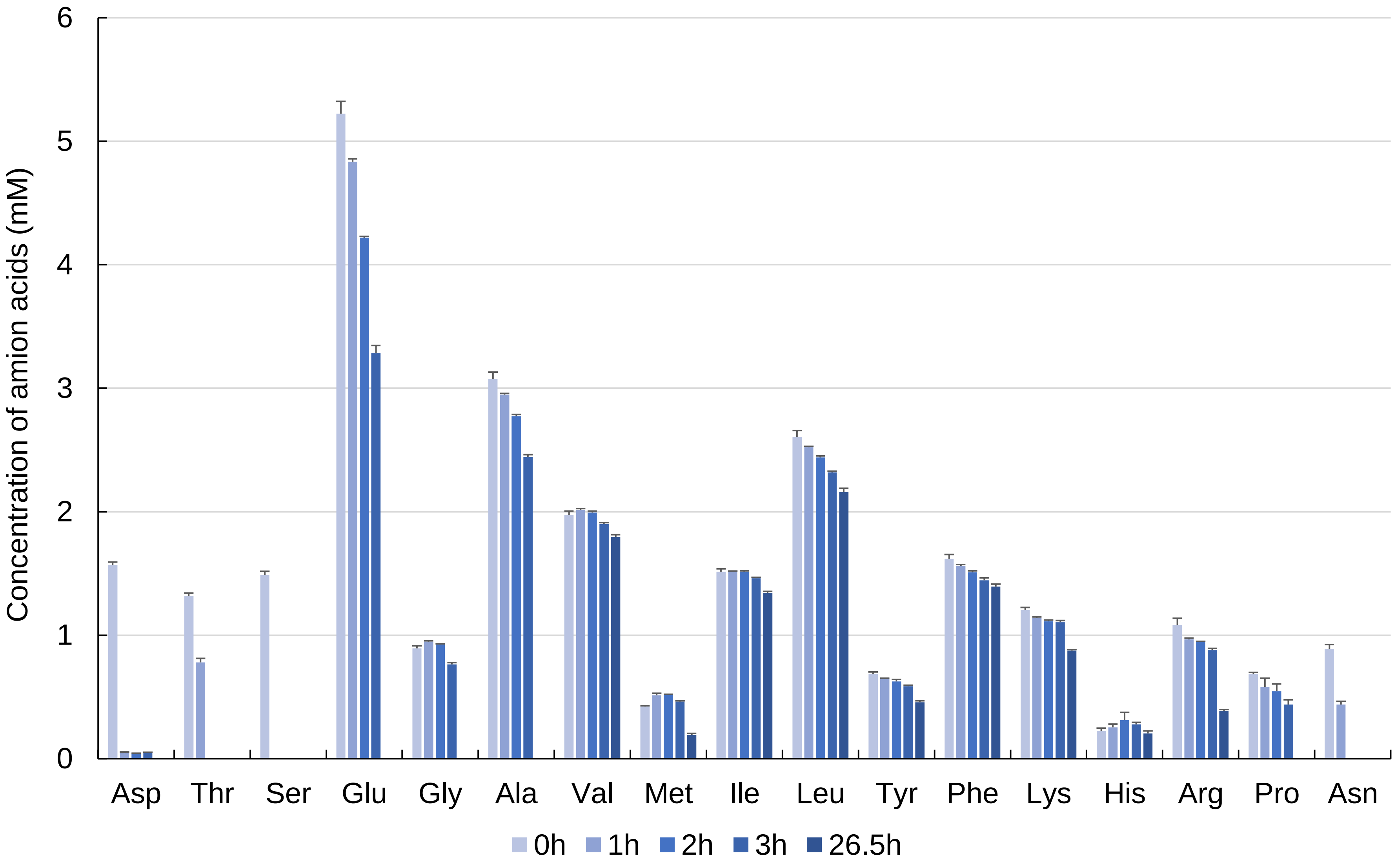


Figure S1. M9-yeast extract medium supplemented with (A) blank, (B) 10 mM glucose, or (C) 50 mM glucose was incubated by *E. coli* DH10B for 1–26.5 hours, and the amino acid concentrations were compared. Values are given as mean ± SD (n=3).

**Fig.S1B**

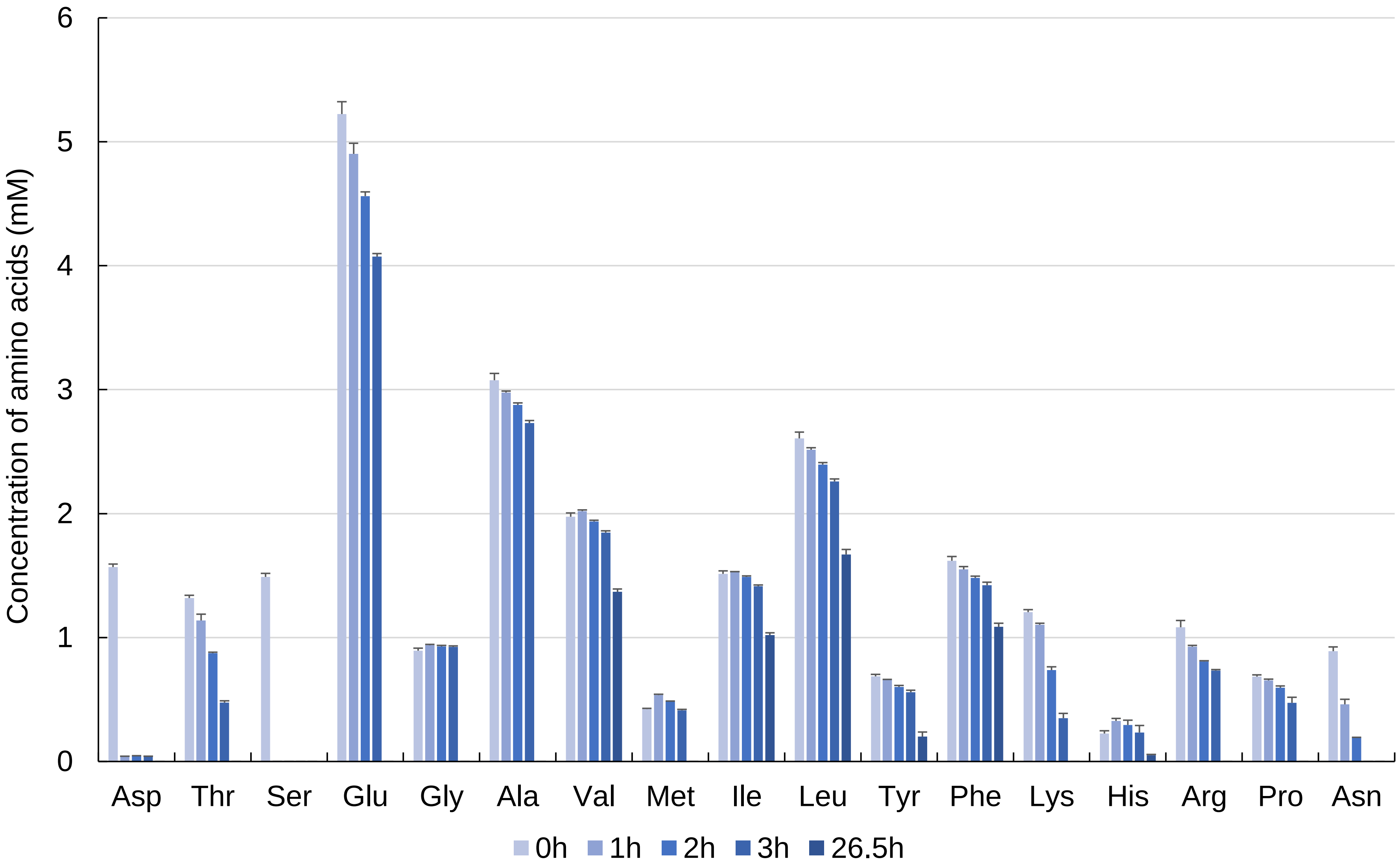

Concentration of amino acids (mM)
6
5
4
3
2
1
0
Asp
Thr
Ser
Glu
Gly
Ala
Val
Met
Ile
Leu
Tyr
Phe
Lys
His
Arg
Pro
Asn
0h
1h
2h
3h
26.5h

**Fig.S1C**

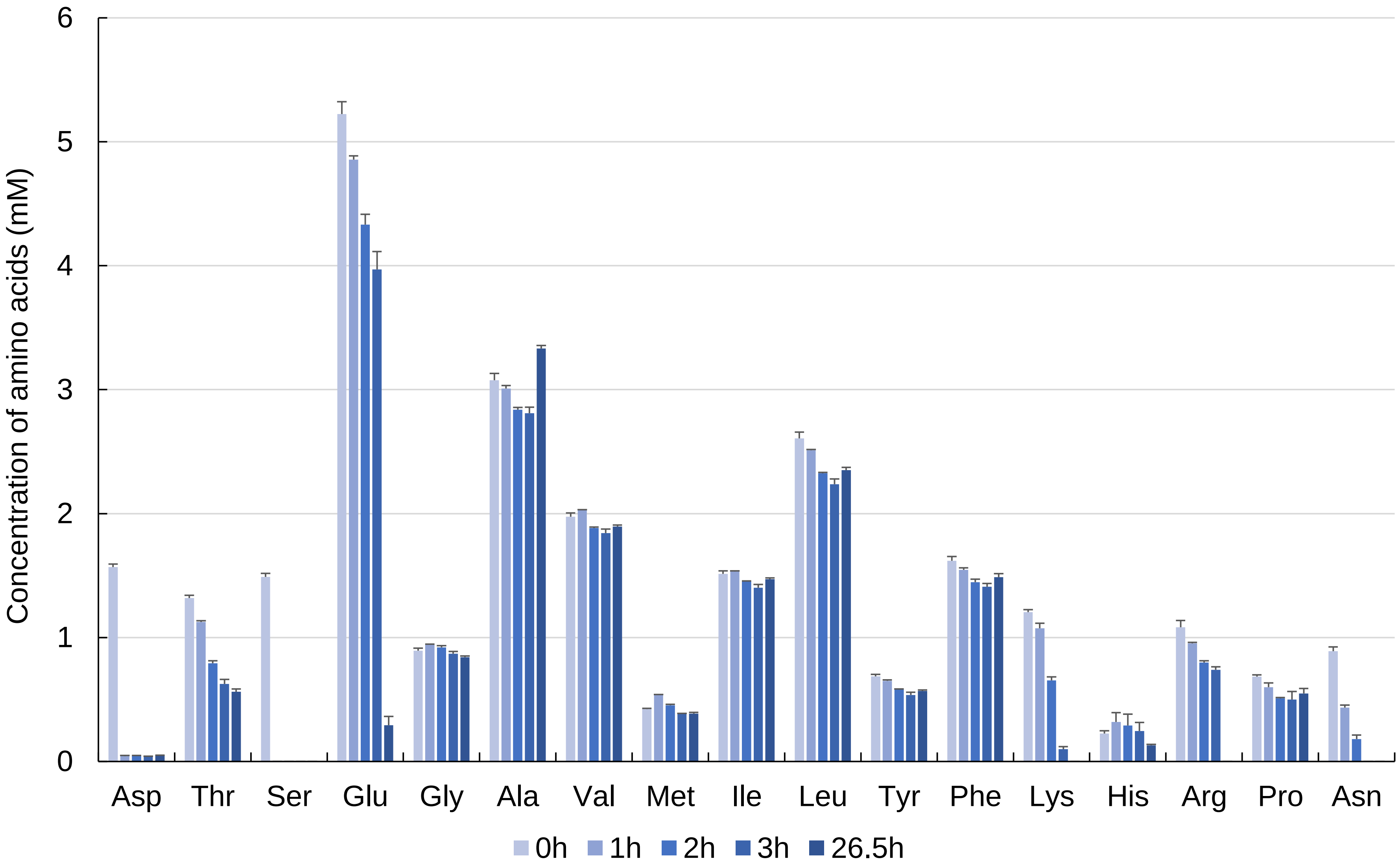
Concentration of amino acids (mM)
6
5
4
3
2
1
0
Asp
Thr
Ser
Glu
Gly
Ala
Val
Met
Ile
Leu
Tyr
Phe
Lys
His
Arg
Pro
Asn
0h
1h
2h
3h
26.5h
